# One-Time Foliar Application and Continuous Resupply via Roots Equally Improved the Growth and Physiological Response of B-Deficient Oilseed Rape

**DOI:** 10.3390/plants10050866

**Published:** 2021-04-26

**Authors:** Anh Quang Dinh, Asif Naeem, Amit Sagervanshi, Karl H. Mühling

**Affiliations:** 1 Institute for Plant Nutrition and Soil Science, Kiel University, Hermann Rodewald Strasse 2, D-24118 Kiel, Germany; aqdinh@plantnutrition.uni-kiel.de (A.Q.D.); anaeem@plantnutrition.uni-kiel.de (A.N.); asagervanshi@plantnutrition.uni-kiel.de (A.S.); 2 Faculty of Agriculture and Forestry, Dalat University, Da Lat City 670000, Lam Dong Province, Vietnam

**Keywords:** boron, *Brassica napus*, B resupply, foliar application, B transporters

## Abstract

Oilseed rape (*Brassica napus* L.) is a high-boron (B)-demanding crop, and initially, normal growing plants might show B deficiency at advanced growth stages on soils with marginal B availability. Hence, we compared the effects of B resupply via roots and leaves on growth and physiological response, and relative expression of B transporters in B-deficient oilseed rape plants. Four-week-old plants initially grown with inadequate B (1 µM B for the first two weeks and 0.25 µM B for the next two weeks) were later grown either as such with 0.25 µM B, with 25 µM B in nutrient solution or foliar sprayed with 7 mL of 30, 60 and 150 mM B solution plant^−1^ as boric acid. Plants grown with 25 µM B in the nutrient solution from the beginning were included as adequate B treatment. Results showed that B resupply to B-deficient plants via roots and leaves (60 mM B) equally improved root and shoot dry matter, but not to the level of plants grown with adequate B supply. Foliar-applied 150 mM B proved toxic, causing leaf burn but not affecting dry matter. Resupply of B via roots increased B concentration in roots and leaves, while leaf-applied B did so only in leaves. Net carbon assimilation had a positive relationship with dry matter accumulation. Except for the highest foliar B level, B resupply via roots and leaves increased the accumulation of glucose, fructose and sucrose in leaves. Boron-deficient plants showed significant upregulation of *BnaNIP5;1* in leaves and roots and of *BnaBOR1;2* in roots. Boron resupply via roots reversed the B-deficiency-induced upregulation of *BnaNIP5;1* in roots, whereas the expression of *BnaBOR1;2* was reversed by both root and foliar B resupply. In leaves, B resupply by both methods reversed the expression of *BnaNIP5;1* to the level of B-adequate plants. It is concluded that B resupply to B-deficient plants via roots and leaves equally but partially corrected B deficiency in *B. napus* grown in hydroponics.

## 1. Introduction

Oilseed rape (*Brassica napus* L.) is one of the major oilseed crops and used worldwide for animal and human nutrition. It is highly sensitive to B deficiency, having B requirements higher than 0.5 mg B kg^−1^ soil [1], and it shows a notable reduction in seed yield and quality under B-deficient conditions [2,3]. Nevertheless, oilseed rape is often cultivated on soils with low or reduced B availability to plants by liming, high soil pH and drought periods during the main growth stages [4,5]. Therefore, a continuous B supply is important throughout the vegetative and reproductive stages for normal growth of plants. Boron deficiency appears often after hot, dry weather because less B can be absorbed by plants as the top soil dries out. The decreased availability probably results from B becoming less mobile in the mass flow to the roots and by polymerization of boric acid [2]. Boron deficiency is considered to inhibit plant growth and reduce yield in oilseed rape [1,6,7]. Physiological responses of the plants to B deficiency relate to the instability of the cell wall and membrane, resulting in the inhibition of the root elongation and expansion of leaves [3,8]. In addition, B deficiency affects carbohydrate metabolism and nucleic acid the synthesis, and thus could lead to a complete inhibition of the absorption and transport of the nutrients in severe cases [2,9]. The photosynthetic rate, transpiration rate, stomatal conductance, leaf gas exchange and intercellular CO_2_ concentration are severely arrested under B deficiency [10].

The B deficiency in plants can be alleviated by the application of B fertilizers, as soil fertilization or foliar spray [1,11,12]. Studies have shown that foliar application of B fertilizers increases crop yields [13] by improving B concentration in leaves and fruiting parts of the plants [14]. A three-year field experiment in Germany showed that foliar application of 250 g B ha^−1^ twice annually, on each occasion together with Epsom salt, led to increased yield of *B. napus* [15]. Jankowski et al. [16] reported that leaf application of B at 150–300 g ha^−1^ to winter oilseed rape significantly improved the nutritional value of the seeds. Recently, Ma et al. [17] reported that foliar application of 500 mg B ha^−1^ during the early flowering stage of canola plants in the field trial showed elevated B concentration and accumulation in straw but this effect was not fully translated to seed yield.

Under conditions of restricted soil B availability, foliar application of B may be more effective than soil application because the foliar-applied B can be directly taken up via leaves and is not fixed in the soil as it happened with the soil application [1,12]. Moreover, for *Brassicaceae* and *Chenopodiaceae* species that have higher B requirements than other crops, fertilization of soils with B can lead to toxicity of this micronutrient to the successive crops [18]. These come up with the assumption that foliar application of B under such conditions is a priority alternative to soil application. However, the efficiency of foliar-applied B to mitigate B deficiency depends on B mobility through the phloem [14,19], which is linked to the possibility of B complexing with sugar alcohols and/or sucrose [20,21,22,23]. Boric acid is the main boron fertilizer, and being an uncharged molecule, it has high permeability through the plasma membranes [24]. The passive diffusion of boric acid is dominated when the available concentration of B is sufficient for the plants [25]. The uptake and transport of B in plants under limited B supply is ensured by NIP5;1, which play an important role in facilitating the diffusion of boric acid across the plasma membrane and BOR1 which facilitate the transport of borate anion from roots to shoots via xylem loading [26,27,28,29]. Recently, Diehn et al. [30], Zhang et al. [31] and Yang et al. [32] mentioned the functions of the specific genes *BnaNIP5;1* and *BnaBOR1;2* involved in the uptake and transport of B in *B. napus*. Although the practice of foliar B application has been used worldwide, little research has been carried out on the comparative physiological and growth responses of B-deficient *B. napus* plants resupplied with B via roots and leaves. Therefore, we investigated the effect of B resupply via roots and foliage on the growth, B concentration in different tissues, physiological behavior and the relative expression of specific genes involved in B uptake and translocation in B-deficient *B. napus* plants under hydroponics condition for two weeks. Different levels of foliar-applied B were compared for their effectiveness and any possible toxicity.

## 2. Results

### 2.1. Roots and Shoots Dry Matters

**Figure 1:**
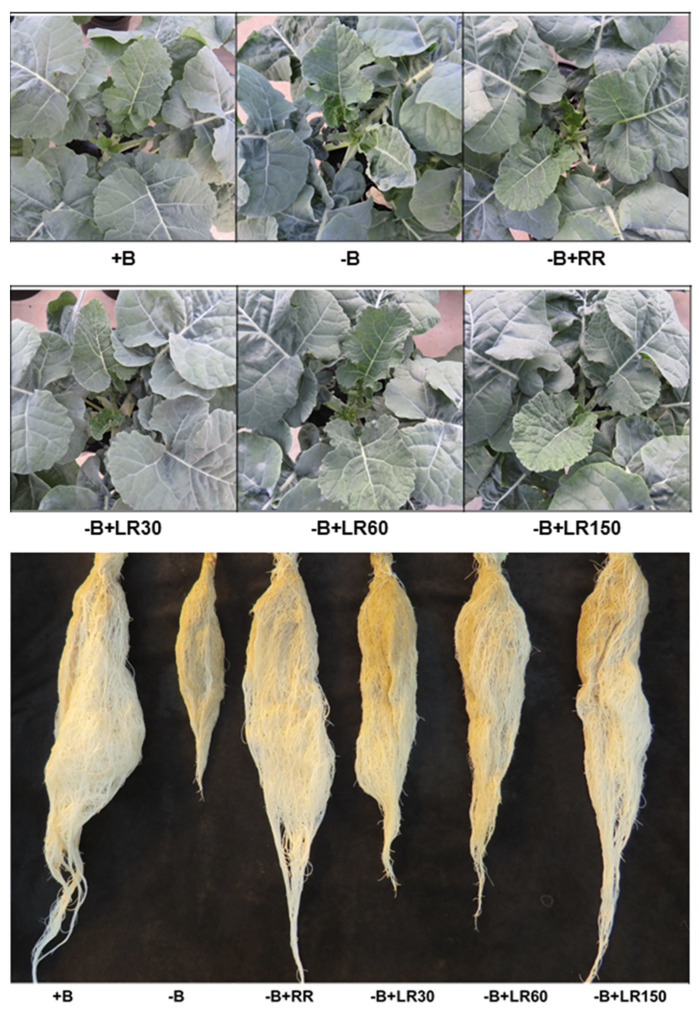
Response of *B. napus* to different B treatments after 42 days of transplantation in the nutrient solution. +B, 25 µM B in NS; −B, 0.25 µM B in NS; −B+RR, 0.25 µM B in NS for the first two weeks and then 25 µM B for the latter two weeks; −B+LR_30_, 0.25 µM B in NS and foliar-applied 30 mM B at two weeks; −B+LR_60_, 0.25 µM B in NS and foliar-applied 60 mM B at two weeks; −B+LR_150_, 0.25 µM B in NS and foliar-applied 150 mM B at two weeks.

Oilseed rape plants grown under B-deficient conditions showed the symptoms of B deficiency both in the foliar mature leaves (rolled down at the margins, remain green) and young leaves (stunted and curled upwards at the margins) (Figure 1). The plants sprayed with 150 mM B solution showed a slight leaf burn at the margins. The growth of B-deficient plants was substantially recovered following B resupply both via roots and leaves.

**Figure 2:**
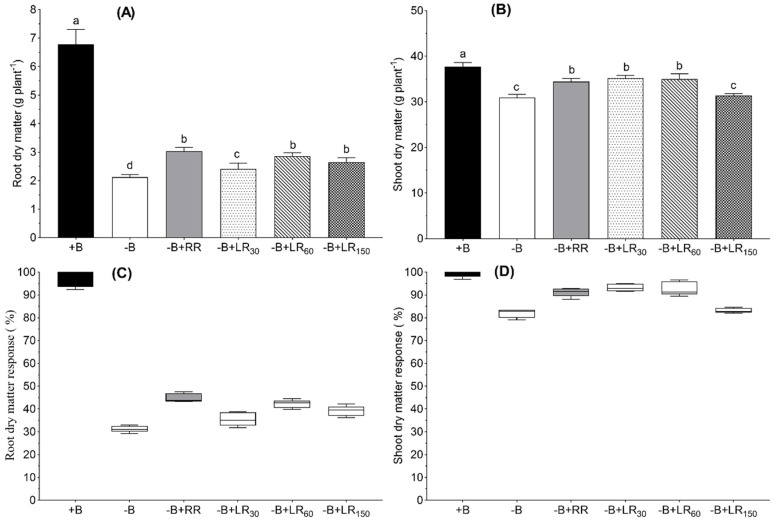
Root (**A, C**) and shoot (**B, D**) dry matters and their responses to different B treatments in nutrient solution grown *Brassica napus* for 42 days. +B, 25 µM B in NS; −B, 0.25 µM B in NS; −B+RR, 0.25 µM B in NS for the first two weeks and then 25 µM B for the latter two weeks; −B+LR_30_, 0.25 µM B in NS and foliar-applied 30 mM B at two weeks; −B+LR_60_, 0.25 µM B in NS and foliar-applied 60 mM B at two weeks; −B+LR_150_, 0.25 µM B in NS and foliar-applied 150 mM B at two weeks. The data ± SE are means of five independent pot replicates. Different letters on bars indicate significant differences between treatments (ANOVA with Duncan’s multiple range test *p* ≤ 0.05).

Root dry matter of −B control plants was only 31% of that produced under +B control (Figure 2A). Boron resupply via the roots to B-deficient plants improved root dry matter by 20% over the −B control (Figure 2A). However, the effect of B resupply via leaves on root dry matter depended upon the B concentration in foliar spray: −B+LR_30_ treatment did not significantly improve the root dry matter whereas −B+LR_60_ and −B+LR_150_ improved root dry matter by 27% and 21%, respectively. Shoots dry matter under −B control was 80% of that obtained under the +B control. Boron root resupply and leaf resupply treatments −B+LR_30_ and −B+LR_60_ significantly improved shoot dry matter and the magnitude of increases over −B control were 9%, 11% and 11%, respectively. Leaf resupply treatment −B+LR_150_ did not affect shoot dry matter as compared to −B control (Figure 2B).

### 2.2. Boron Concentration

**Figure 3:**
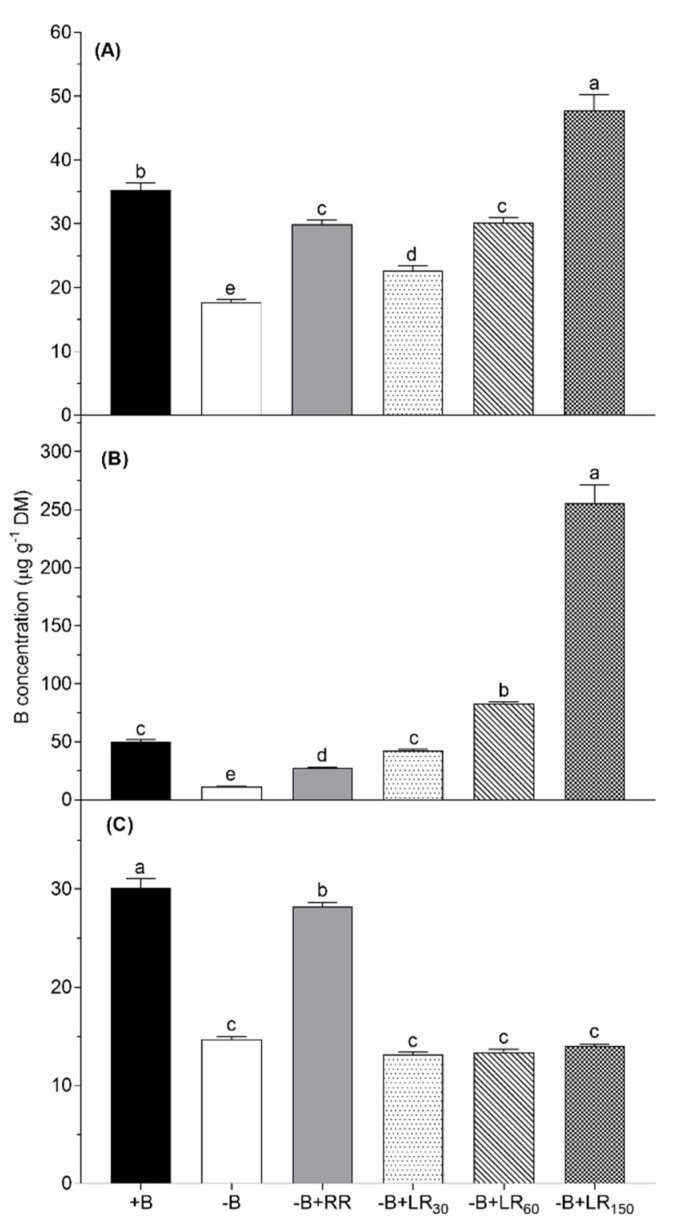
Boron concentration in young leaves (**A**), mature leaves (**B**) and roots (**C**) of *Brassica napus* grown in nutrient solution under different B treatments for 42 days. +B, 25 µM B in NS; −B, 0.25 µM B in NS; −B+RR, 0.25 µM B in NS for the first two weeks and then 25 µM B for the latter two weeks; −B+LR_30_, 0.25 µM B in NS and foliar-applied 30 mM B at two weeks; −B+LR_60_, 0.25 µM B in NS and foliar-applied 60 mM B at two weeks; −B+LR_150_, 0.25 µM B in NS and foliar-applied 150 mM B at two weeks. The data ± SE are means of five independent pot replicates. Different letters on bars indicate significant differences between treatments (ANOVA with Duncan’s multiple range test *p* ≤ 0.05).

The highest B concentration in the roots (30 mg kg^−1^ DM) was recorded for +B control plants, while in −B control plants, the root B concentration was only 42% of what was recorded for +B control (Figure 3A). Boron resupply via roots (−B+RR) to B-deficient plants increased B concentration up to 90% of what was recorded for +B control plants. Boron resupply via leaves did not affect the B concentration in roots. In mature leaves, both root and foliar resupply improved B concentration, and increase with the foliage resupply was dose-dependent and higher than the root resupply (Figure 3B). Moreover, B concentration in the mature leaves with root resupply could not reach the level of +B control, which was achieved even with the lowest dose of foliage resupply. In young leaves, the B resupply treatments increased B concentration in the same manner as they did for mature leaves (Figure 3C).

However, B concentration in young leaves reached the level of or higher than +B control plants with only the highest dose (150 mM B) of foliage resupply. Boron root resupply and foliar resupply with 60 mM B had similar B concentrations in the young leaves, and these were 47% higher than the −B control plants.

### 2.3. Photosynthetic and Transpiration Rates

**Figure 4:**
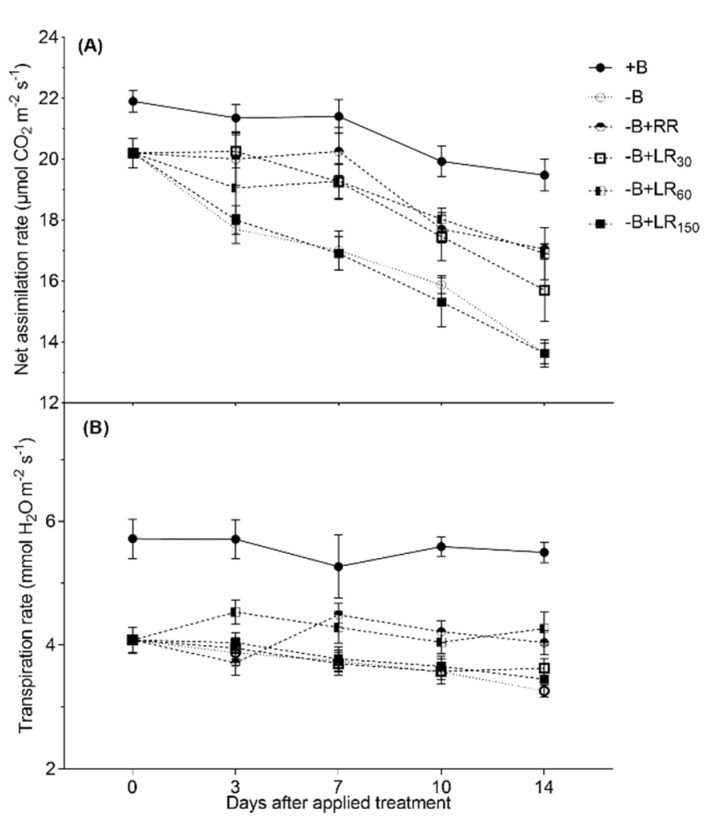
Changes in net CO_2_ assimilation rate (**A**; µmol CO_2_ m^−2^ s^−1^) and transpiration rate (**B**; µmol H_2_O m^−2^ s^−1^) of *Brassica napus* grown in nutrient solution under different B treatments for 42 days. +B, 25 µM B in NS; −B, 0.25 µM B in NS; −B+RR, 0.25 µM B in NS for the first two weeks and then 25 µM B for the latter two weeks; −B+LR_30_, 0.25 µM B in NS and foliar-applied 30 mM B at two weeks; −B+LR_60_, 0.25 µM B in NS and foliar-applied 60 mM B at two weeks; −B+LR_150_, 0.25 µM B in NS and foliar-applied 150 mM B at two weeks. The data ± SE are means of four independent pot replicates. Different letters on bars indicate significant differences between treatments (ANOVA with Duncan’s multiple range test *p* ≤ 0.05).

The photosynthetic rate of the B-deficient plants showed a significant decline from 20 to 13 μmol CO_2_ m^−2^ s^−1^ over a period of two weeks after treatment application (Figure 4A). However, during the same period, a negative change of only 2 μmol CO_2_ m^−2^ s^−1^ was recorded for +B control plants. Boron resupply to the B-deficient plants via roots and via leaves with 30 and 60 mM B solution did not affect the photosynthetic rate of *B. napus* after 7 days of treatment application; however, the assimilation rate continued to decrease after 14 days of the treatment period. Photosynthetic rates with neither B resupply via the roots nor via the leaves could reach the level of +B control plants.

Transpiration rates of the control plants did not differ between the treatments significantly throughout the whole measuring period (Figure 4B). Boron resupply via roots or leaves with 60 mM B solution slightly increased the transpiration rate compared to the −B control. The plants resupplied via foliage with 30 and 150 mM B solution did not improve the transpiration rate during the two-week treatment period.

### 2.4. Soluble Sugars

**Figure 5:**
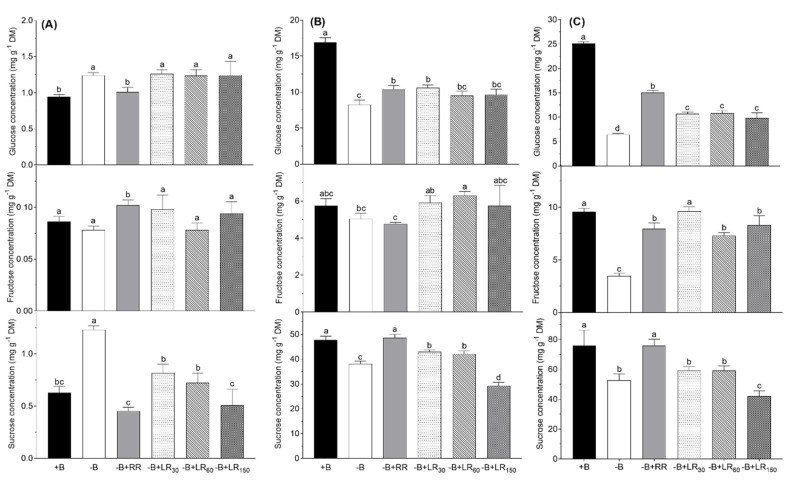
Glucose, fructose and sucrose concentrations in the roots (**A**), mature leaves (**B**) and young leaves (**C**) of *Brassica napus* grown in nutrient solution under different B treatments for 42 days. +B, 25 µM B in NS; −B, 0.25 µM B in NS; −B+RR, 0.25 µM B in NS for the first two weeks and then 25 µM B for the latter two weeks; −B+LR_30_, 0.25 µM B in NS and foliar-applied 30 mM B at two weeks; −B+LR_60_, 0.25 µM B in NS and foliar-applied 60 mM B at two weeks; −B+LR_150_, 0.25 µM B in NS and foliar-applied 150 mM B at two weeks. The data ± SE are means of five independent pot replicates. Different letters on bars indicate significant differences between treatments (ANOVA with Duncan’s multiple range test *p* ≤ 0.05).

Concentrations of soluble sugars in both the mature leaves and young leaves were by many folds higher than those recorded in the roots (Figure 5). Boron deficiency (−B) led to a substantial increase in glucose and sucrose concentration in roots as compared to +B control plants and those resupplied via roots. In foliar B resupplied plants, glucose concentration remained the same as it was in −B control plants, but sucrose concentration decreased in the dose-dependent manner, reaching the level of +B plants with the highest dose of B foliar spray. In the mature and young leaves, B deficiency led to a decline in the concentrations of all sugars, and the decline was more drastic in young leaves. Boron resupply via roots increased glucose and sucrose concentrations in the mature leaves while it improved the concentrations of all the soluble sugars in young leaves. In most of the cases, the improvement in the concentration of soluble sugars was maximum at the lowest rate of foliar B resupply, which progressively decreased with an increase in the rate of application, and became nonsignificant or negative in some cases.

Both in mature leaves and young leaves, B resupply via foliage produced a higher increase in the concentration of fructose, whereas reverse response was recorded for sucrose concentration.

### 2.5. Relative Expression of BnaBOR1;2 and BnaNIP5;1 in Roots and Leaves

**Figure 6:**
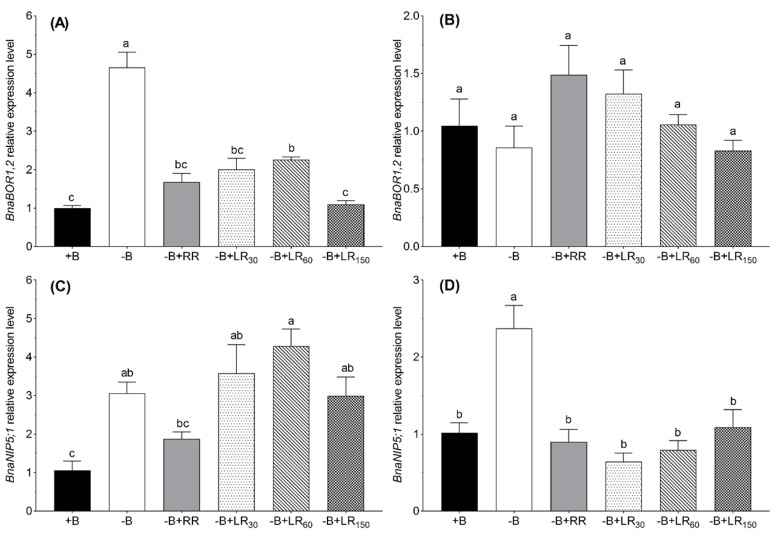
The fold change in the relative expression of *BnaBOR1;2* and *BnaNIP5;1* gene in roots (**A**,**C**) and leaves (**B**,**D**) of *Brassica napus* grown in nutrient solution under different B treatments for 42 days. +B, 25 µM B in NS; −B, 0.25 µM B in NS; −B+RR, 0.25 µM B in NS for the first two weeks and then 25 µM B for the latter two weeks; −B+LR_30_, 0.25 µM B in NS and foliar-applied 30 mM B at two weeks; −B+LR_60_, 0.25 µM B in NS and foliar-applied 60 mM B at two weeks; −B+LR_150_, 0.25 µM B in NS and foliar-applied 150 mM B at two weeks. Bars represent means of three biological replicates, technically replicated two times ± SE. Different letters on bars indicate significant differences between treatments (ANOVA with Duncan’s multiple range test *p* ≤ 0.05).

In order to study whether resupply B in the nutrient solution or foliar-applied affects the transport pathway of B under B deficiency, the expression of genes potentially involved in B uptake and transport in *B. napus* plants were investigated. As compared to B-sufficient (+B control) plants, B deficient plants (−B control) showed significant upregulation of *BnaNIP5;1* in the roots (3-fold) and leaves (1.5-fold), and of *BnaBOR1;2* in only roots (4.5-fold) (Figure 6). In the roots, the B-deficiency-induced upregulation of both *BnaBOR1;2* and *BnaNIP5;1* was significantly reversed by B resupply via roots, but the expression levels were still higher than the +B control plants. Similarly, B resupply via foliage reversed the B-deficiency-induced upregulation of *BnaBOR1;2* in roots and the expression reached the level of +B control plants at the highest rate of foliar B application. However, B resupply via foliage could not reverse the B-deficiency-induced upregulation of *BnaNIP5;1* in roots. In leaves, both methods of B resupply reversed the B-deficiency-induced upregulation of *BnaNIP5;1* to the level of +B control plants. Interestingly, the relative expression of *BnaBOR1;2* in the leaves was affected neither by B deficiency nor by B resupply to the B-deficient plants.

## 3. Discussion

In this study, we observed B deficiency symptoms (rolled down at the margins, remain green) in the leaves of B-deficient *B. napus* plants (Figure 1). In accordance with this and as it was expected, B-deficient plants produced smaller amounts of root and shoot dry matter than B-sufficient plants (Figure 2). This is in agreement with the common fact that plants grown under B-deficient conditions show reduced yield [1,16,33]. Boron concentration in the leaves of B-deficient plants was also close to or below the critical concentration of B for *B. napus* plants [34]. On the other hand, B resupply both via roots and foliage to the B-deficient plants increased root and shoot dry matter of *B. napus* (Figure 2). Improvement in dry matter yield was concomitant with the increase in B concentration in the leaves and roots (Figure 3) in the roots resupply treatment. Some previous studies showed that foliar B application could improve B availability in leaves and increase the B concentration in leaves [14,17,33,35,36,37]. The fact that foliar B resupply increased the concentration of B in treated leaves as well as young leaves (Figure 3B and C) implies that foliar-applied B can be transported from mature leaves to young leaves or the reproductive organs via the phloem [14,18,38,39,40,41,42]. According to Stangoulis et al. [23], B has moderate phloem mobility in oilseed rape, since limited amounts of borate can be translocated to the young tissues through binding with sucrose. In this study, B resupply via roots and leaves to B-deficient plants increased shoot dry matter more than root dry matter (Figure 2), depicting that root growth is more sensitive to B deficiency than shoot growth. However, resupply of B via the leaves to B-deficient oilseed rape plants did not increase B concentration in the roots as compared to the −B control treatment. These results are in agreement with the results from Asad et al. [33], who found that leaf application of B to B-deficient sunflower plants did not improve B concentration in the roots even with the highest B content of sprayed solution (1200 mM B). The possible reason for this could be the dilution effect since B content in the roots of the −B treatment was not significantly different to that of foliage resupply treatments (data not shown) and the root dry matter of foliage treatments increase together with the increase of B resupply. The other reason could be the recycling of B from the roots to the shoot via xylem loading [14]. Our result also showed that B concentration in mature leaves of foliar−B-resupplied to B-deficient plants reached the level of +B control plants even with 30 mM B, and the concentration further increased with the increasing foliar-applied B level. However, B concentration in the young leaves of 30 mM B and 60 mM B treatments did not reach the level of +B control plants. Our findings are in agreement with the result from Orlovius [15] and Stangoulis et al. [23], who demonstrated that B has moderate mobility in oilseed rape. Thus, it could be interpreted that obtaining a sufficient concentration of B in young leaves requires a relatively higher dose of B in the foliar spray. In this regard, the required level of B leaf application (150 mM B) showed a significant decrease in shoot dry matter and photosynthesis rate. The highest foliar B concentrations in the present experiment might have led to high free and unbound B in the leaves which became toxic for the plants. Boron concentration in leaves with this foliar treatment was close to the toxic levels (above 200 µg g^−1^) of B in *B. napus* [43] which implies impracticability of using this B level for foliar B application. A large surface area of the leaves together with the high amount of B in the foliar spray (150 mM B) would have led to the over-accumulation of B in the leaves. 

In our study, B-deficient plants showed a significant decrease in photosynthesis whereas B resupply via roots (25 µM B) and leaves (30 and 60 mM B solution) resulted in increased photosynthetic and transpiration rates (Figure 3). Photosynthesis was improved by 15% with both B resupply via roots and foliar application of 60 mM B solution, which explains the improvement in roots and shoot dry matters under these treatments. Hossain et al. [44] reported that the photosynthetic rate of oilseed rape plants was increased when soil was fertilized with 9 kg B ha^−1^ in the field experiment. This increase in transpiration rate by B resupply could have been caused by the increase in the number and functioning of stomata under B-sufficient conditions [10,14,45]. Foliar spray of 150 mM B solution inhibited shoot growth, photosynthesis rate and transpiration rate as compared to the +B control treatment. In addition, we might speculate that in resupply of B via root or leaves, the recovery of photosynthesis might be due to the plant having a greater capability to expand the leaves and accumulate more chlorophyll in the leaves [46].

Boron deficiency and resupply had different effects on the concentration of soluble sugars in the plant tissues. Glucose, fructose and sucrose concentrations showed a significant decrease in the leaves of *B. napus* in −B treatment as compared to +B control (Figure 4). In line with the current study, Hegazi et al. [47] and Zhao and Oosterhuis [48] reported that concentrations of sugars in the leaves decreased under B deficiency. Resupply via the roots in nutrient solution and leaves as foliar spray (only 30 and 60 mM B solution) to B-deficient plants led to increased glucose, fructose and sucrose concentrations in leaves. This is well-justified by the improved photosynthetic rate under these B resupply treatments. In this case, roots and leaf application of B to B-deficient plants had beneficial effects on shoot growth but it did not appear under the highest level of foliar B treatment (150 mM B solution).

Although it is well-known that *BnaNIP5,1* and *BnaBOR1;2* genes are ubiquitously expressed and have a significant role in B absorption and translocation in *B. napus* [30,32,49,50], the effect of roots or leaf resupply on the expression of these genes in B-deficient plants was still lacking. We found that the relative expressions of both genes in the roots of −B control plants were significantly upregulated (Figure 6), confirming that the B in nutrient solution was not sufficient for plant growth under this treatment. Since B concentration in the roots and leaves was close to the critical level for B deficiency, *BnaNIP5,1* channel and *BnaBOR1;2* transporters were highly upregulated to enhance the absorption and transport of boric acid into the root cell and xylem to meet the B demands of plants [25,27]. Boron resupply via roots to B-deficient plants resulted in downregulation of *BnaNIP5,1* and *BnaBOR1;2* in the roots and reached close to the level of +B control plants (Figure 6), suggesting that B resupply via roots is sufficient for passive diffusion of boric acid via the plasma membrane into the roots and transport to the shoot thought transpiration stream. This agrees with the fact that resupply of B via the roots to the B-deficient plants partially retrieved the root and leaf B concentration, root and shoot dry matter and physiological attributes, as compared to the +B control plants. Interestingly, the relative expression of *BnaNIP5,1* in the roots of foliage treatments was upregulated even at the highest B sprayed (150 mM B), whereas the relative expression of *BnaBOR1;2* was not significantly different as compared to the +B control, except for the foliage treatment resupply with 150 mM B. In addition, the expression levels of *BnaNIP5,1* and *BnaBOR1;2* were mainly upregulated in the roots of B-deficient *B. napus* plants [30,51]. The data from this study confirm the role of *BnaNIP5,1* and *BnaBOR1;2* in the uptake and distribution of B in response to B supply. However, a full reversal of the B-deficiency-induced upregulation in the relative expression of *BnaNIP5,1* in leaves by B resupply via both the roots and leaves (Figure 6) might be explained by high B concentration in the treated leaves, which was enough to support the phloem re-translocation needs of the plants by passive diffusion of B through the plasma membrane [14,22,52].

## 4. Materials and Methods

### 4.1. Plant Cultivation and Treatment Application

Winter oilseed rape (*Brassica napus* L. cv. Alpaga, Norddeutsche Pflanzenzucht Lembke (NPZ), Hohenlieth, Germany) was grown hydroponically in the greenhouse at day/night temperatures of 22 °C/18 °C and the photoperiod from 8:00 to 22:00 with light intensity of 350 µM photon m^−2^ s^−1^ (recorded by a light meter, Li-198, Lincoln, NE, USA). Seeds were soaked in aerated 1 mM CaSO_4_ solution for 24 h, germinated on filter paper in darkness, and then the seedlings were exposed to light. Six-day-old, uniform-sized seedlings were transferred to plastic pots (2 plants per pot) containing 10 L of one-quarter-strength nutrient solution. The strength of the nutrient solution was increased to one-half- and full-strength on day 4 and 8 days of transplantation, respectively. From day 8 onward, only one plant was retained in each pot containing the full-strength nutrient solution. The composition of the full-strength nutrient solution was as follows: 2.0 mM Ca(NO_3_)_2_, 0.5 mM K_2_SO_4_, 0.25 mM KH_2_PO_4_, 0.325 mM MgSO_4_, 50 µM NaCl, 1 µM H_3_BO_3_, 2 µM MnSO_4_, 0.4 µM ZnSO_4_, 0.4 µM CuSO_4_, 0.1 µM Na_2_MoO_4_ and 40 µM Fe-EDTA. The nutrient solution was replaced every fourth day and the pH of the nutrient solution was adjusted to 6 ± 0.2 every second day using 0.1 M HCl. To avoid B contamination, double deionized water (18.2 MΩ) was used to prepare the nutrient solution throughout the study.

For the first two weeks, the plants were grown at 1 µM B (sufficient to support plant growth at this stage), and for the latter two weeks, B concentration in nutrient solution was reduced to 0.25 µM. Plants showed severe B deficiency after the fourth week of growth. Boron-deficient, four-week-old plants (each having seven leaves) were treated as follows: 1st group of plants was allowed to grow as such with 0.25 µM B in the nutrient solution (negative control, −B), 2nd group was resupplied with 25 µM B (as H_3_BO_3_) in the nutrient solution (−B+RR), while the 3rd, 4th and 5th groups were foliar-sprayed with 7 mL of 30 (−B+LR_30_), 60 (−B+LR_60_) and 150 (−B+LR_150_) mM B (as H_3_BO_3_, equivalent to 2.25, 4.5 and 11.3 mg B plant^−1^, respectively). Each B spray solution was adjusted to the pH 5.3 using 0.1 N NaOH. A positive control (+B), supplied with 25 µM H_3_BO_3_ in the nutrient solution from the beginning, was also included among the treatments. Each treatment had ten independent pot replications. Since the temperature of the greenhouse was low in the morning, foliar treatments were realized at this time to avoid immediate evaporation of the applied spray solution. Silwet^®^ Gold (0.1%) was added as a wetting agent to enhance adherence of the spray solution to leaves. The pots were covered with polyethylene sheets while spraying the plants to avoid the contamination of the nutrient solution.

### 4.2. Gas Exchange Measurements

Gas exchange measurements were carried out by a portable gas exchange system (LI-6400XT, LI-COR Biosciences, Lincoln, NE, USA). Photosynthetic photon flux density, provided by a red/blue LED light source, was adjusted to 1000 µmol m^−2^ s^−1^ and ambient CO_2_ concentration to 400 µmol mol^−1^ by CO_2_ injection. The first measurement was taken just before applying B treatments, and then every 4th day until the harvesting of plants. Measurements were taken from 10 a.m. to 16 p.m. on a central 6 cm^2^ leaf segment of the 6th leaf.

### 4.3. Harvesting and Sample Preparation

Plants were harvested two weeks after applying B treatments, separated into mature leaves (lower seven leaves), young leaves (untreated leaves) and roots, and washed with deionized water. Plant samples from one set of five replicates were oven-dried at 65 °C for 72 h and used for recording dry matters and analyzing minerals and sugars. The second set of five replicates was frozen in liquid N and stored at −80 °C for transporters’ measurements. The dried tissue samples were ground to fine powder using a ball-mill grinder.

### 4.4. Determination of Boron

The dried samples (approx. 200 mg each) were digested with 10 mL of 69% HNO_3_ (ROTIPURAN Supra for ICP, 69%) in a closed-vessel 1800-watt microwave digestion system (MARS 6 Xpress, CEM Corporation, Matthews, USA) adjusted to the following conditions: 2 min at 100 °C, 1 min at 120 °C, 20 min at 180 °C and 20 min cooling time. Afterwards, the digested samples were diluted to 100 mL and stored at 4 °C until further analysis. The concentration of B in the roots and shoots were determined by ICP-MS (Inductively Coupled Plasma Mass Spectroscopy, Agilent 7700, Agilent Technologies Inc., Santa Clara, CA, USA). To avoid B contamination from glassware, only plasticware were used throughout the experimentation and during B analysis.

### 4.5. Determination of Soluble Sugars

Soluble sugars were extracted from 30 mg of dry material using 1.5 mL of double deionized water. The samples were placed in a shaking bath (Medingen SWB20, Dresden, Germany) preheated to 100 °C for 5 min. Thereafter, the samples were cooled on ice for 30 min and then centrifuged for 10 min at 12,000 rpm at 4 °C. The supernatants were collected and diluted 10 times. The samples were mixed one more time with the vortex (5 s) and centrifuged (12,000 rpm, 5 min). Samples were cleaned-up with chloroform and run over C18 columns (Strata^®^. 8B-S001-DAK, Phenomex, Torrance, CA, USA). Sucrose, glucose and fructose concentrations were measured by anion exchange chromatography using an ICS-5000 system equipped with a Carbo Pac PA-100 column and an integrated amperometric detector (Dionex, Sunnyvale, CA, USA).

### 4.6. Primer Design and Sanger Sequencing

To investigate the mRNA transcript level of *BnaBOR1;2* and *BnaNIP5;1* transporters, primers were designed from sequences available on NCBI database for *BnaBOR1;2* (ID GU827643) and *BnaNIP5;1* (KT899999) of *Brassica napus*. Primer3plus (http://www.bioinformatics.nl/cgi-bin/primer3plus/primer3plus.cgi/, accessed on 8 Octobor 2019), and primer−Blast (https://www.ncbi.nlm.nih.gov/tools/primer-blast/index.cgi, accessed on 8 Octobor 2019) software were used for primer designing. Screened primer characteristics were checked and evaluated in silico by the online tool OligoCalc (http://biotools.nubic.northwestern.edu/OligoCalc.html, accessed on 8 Octobor 2019) and multiple primer analyzer tools provided by Thermo Fisher. All primer pairs were purchased from Eurofins Genomics (Ebersberg, Germany). The details of the primers are listed in Table S1. Furthermore, amplification of the correct gene was confirmed by the sequencing of the amplicon (Sanger sequencing, Instituts für Klinische Molekularbiologie, Kiel).

### 4.7. RNA Extraction, Reverse Transcription and Real-Time Quantitative PCR

Plant samples from the 2nd set of cultivation previously frozen in liquid nitrogen and stored at −80 °C were used for RNA extraction. Total RNA was isolated from the powdered shoot and root material with TRIZOL reagent (Invitrogen, CA, USA) according to the manufacturer’s protocol. The amount of RNA was quantified using a NanoDrop spectrophotometer (ND1000, Thermo Fisher Scientific, Massachusetts, USA), and purity was checked by gel electrophoresis. The cDNA was synthesized following the manufacturer’s instructions in the Verso^TM^ cDNA kit from Thermo Fisher Scientific as described in detail by Dinh et al. [50].

Quantitative RT-PCR was conducted by PowerUp™ SYBR™ Green Master Mix (Applied Biosystems) with family-specific conserved primers (Table S1) on CFX96 Real-Time System (Bio-Rad Laboratories GmbH, München, Germany). For each reaction, total volume was 20 μL containing 100 nM of each primer and 2 μL of diluted cDNA templates and the sequence of cycling conditions was as follows: 95 °C for 3 min, followed by 40 cycles of 95 °C for 10 s, 60 °C for 30 s, 72 °C for 30 s, followed by 95 °C for 10 min. Three biological replicates and two technical replicates were used for each treatment. Transcript levels of gene were normalized with endogenous control (*BnaActin* rapeseed actin gene, GenBank Accession No. AF111812)) [53], and the fold expression changes of target mRNAs were determined using the 2^–ΔΔCt^ method [54].

### 4.8. Statistical Analysis

The data were statistically analyzed using SPSS software version 22.0 (IBM Corp., Armonk, NY, USA). All of the data were tested for normality distribution with Shapiro–Wilk test before analysis. Reported data values in the figures are mean ± SE of five indepedent pot replicates. Effects of the treatments were tested using one way ANOVA and following Duncan’s multiple range test of six different groups at *p* ≤ 0.05. GraphPad Prism 8 was used for making graphs.

## 5. Conclusions

It is concluded that under controlled conditions, both root and foliar resupply of B to B-deficient oilseed rape plants can partially retrieve the B-deficiency-induced loss in dry matter production, physiological functioning and relative expression of specific B transporters. Spray solution with 60 mM B concentration can be used to get the optimum recovery response without any toxicity to the foliage of oilseed rape plants. One-time foliar resupply of B to B-deficient oilseed rape plants significantly increased B concentration in the leaves and retrieved physiological response, as did the continuous resupply via roots throughout the two-week period of growth. However, a partial retrieval of growth with individual root or foliar resupply to B-deficient plants suggests the need for dual resupply both via roots and foliage to achieve complete growth recovery of the deficient plants.

## Data Availability

The data that support the findings of this study are available from the corresponding author upon reasonable request.

## Supplementary Materials

The following are available online at https://www.mdpi.com/article/10.3390/plants10050866/s1, Table S1: Primers of *BnaBOR1;2* and *BnaNIP5;1* designed for real-time PCR.

## References

[1] Shorrocks V.M. (1997). The occurrence and correction of boron deficiency. Plant Soil.

[2] Marschner P. (2012). Marschner’s Mineral. Nutrition of Higher Plants.

[3] Dell B., Huang L.B. (1997). Physiological response of plants to low boron. Plant Soil.

[4] Wimmer M.A., Goldberg S., Gupta U.C., Barker A., Pilbeam D. (2015). Boron. Handbook of Plant Nutrition.

[5] Brown P.H., Shelp B.J. (1997). Boron mobility in plants. Plant Soil.

[6] Zhang D., Zhao H., Shi L., Xu F. (2014). Physiological and genetic responses to boron deficiency in Brassica napus: A review. Soil Sci. Plant Nutr..

[7] Eggert K., von Wiren N. (2016). The role of boron nutrition in seed vigour of oilseed rape (*Brassica napus* L.). Plant Soil.

[8] Matoh T. (1997). Boron in plant cell walls. Plant Soil.

[9] Brown P.H., Bellaloui N., Wimmer M.A., Bassil E.S., Ruiz J., Hu H., Pfeffer H., Dannel F., Romheld V. (2002). Boron in plant biology. Plant Biol..

[10] Wimmer M.A., Eichert T. (2013). Review: Mechanisms for boron deficiency-mediated changes in plant water relations. Plant Sci..

[11] Fageria N.K., Filho M.P.B., Moreira A., Guimarães C.M. (2009). Foliar Fertilization of Crop Plants. J. Plant Nutr..

[12] Fernández V., Sotiropoulos T., Brown P. (2013). Foliar Fertilization—Scientific Principles and Field Practices.

[13] Noreen S., Fatima Z., Ahmad S., Ashraf M. (2018). Foliar application of micronutrients in mitigating abiotic stress in crop plants. Plant Nutrients and Abiotic Stress Tolerance.

[14] Eichert T., Goldbach H.E. (2010). Transpiration rate affects the mobility of foliar-applied boron in *Ricinus communis* L. cv. Impala. Plant Soil.

[15] Orlovius K. (2001). Effect of foliar fertilisation with magnesium, sulfur, manganese and boron to sugar beet, oilseed rape, and cereals. Plant Nutrition.

[16] Jankowski K.J., Hulanicki P.S., Krzebietke S., Zarczynski P., Hulanicki P., Sokolski M. (2016). Yield and quality of winter oilseed rape in response to different systems of foliar fertilization. J. Elem..

[17] Ma B.-L., Zheng Z., Whalen J.K., Caldwell C., Vanasse A., Pageau D., Scott P., Earl H., Smith D.L. (2019). Uptake and nutrient balance of nitrogen, sulfur, and boron for optimal canola production in eastern Canada. J. Plant Nutr. Soil Sci..

[18] Shelp B.J., Vivekanandan P., Vanderpool R.A., Kitheka A.M. (1996). Translocation and effectiveness of foliar-fertilized boron in broccoli plants of varying boron status. Plant Soil.

[19] Brown P.H., Bassil E. (2011). Overview of the acquisition and utilization of boron, chlorine, copper, manganese, molybdenum, and nickel by plants and prospects for improvement of micronutrient use efficiency. The Molecular and Physiological Basis of Nutrient Use Efficiency in Crops.

[20] Reid R. (2014). Understanding the boron transport network in plants. Plant Soil.

[21] Will S., Eichert T., Fernández V., Müller T., Römheld V. (2012). Boron foliar fertilization of soybean and lychee: Effects of side of application and formulation adjuvants. J. Plant Nutr. Soil Sci..

[22] Will S., Eichert T., Fernández V., Möhring J., Müller T., Römheld V. (2011). Absorption and mobility of foliar-applied boron in soybean as affected by plant boron status and application as a polyol complex. Plant Soil.

[23] Stangoulis J., Tate M., Graham R., Bucknall M., Palmer L., Boughton B., Reid R. (2010). The mechanism of boron mobility in wheat and canola phloem. Plant Physiol..

[24] Dordas C., Brown P.H. (2000). Permeability of boric acid across lipid bilayers and factors affecting it. J. Membr. Biol..

[25] Miwa K., Fujiwara T. (2010). Boron transport in plants: Co-ordinated regulation of transporters. Ann. Bot..

[26] Takano J., Wada M., Ludewig U., Schaaf G., von Wiren N., Fujiwara T. (2006). The Arabidopsis major intrinsic protein NIP5;1 is essential for efficient boron uptake and plant development under boron limitation. Plant Cell.

[27] Takano J., Miwa K., Fujiwara T. (2008). Boron transport mechanisms: Collaboration of channels and transporters. Trends Plant Sci..

[28] Takano J., Noguchi K., Yasumori M., Kobayashi M., Gajdos Z., Miwa K., Hayashi H., Yoneyama T., Fujiwara T. (2002). Arabidopsis boron transporter for xylem loading. Nature.

[29] Yoshinari A., Takano J. (2017). Insights into the Mechanisms Underlying Boron Homeostasis in Plants. Front. Plant Sci..

[30] Diehn T.A., Bienert M.D., Pommerrenig B., Liu Z.J., Spitzer C., Bernhardt N., Fuge J., Bieber A., Richet N., Chaumont F. (2019). Boron demanding tissues of Brassica napus express specific sets of functional Nodulin26-like Intrinsic Proteins and BOR1 transporters. Plant J..

[31] Zhang Q., Chen H.F., He M.L., Zhao Z.Q., Cai H.M., Ding G.D., Shi L., Xu F.S. (2017). The boron transporter BnaC4.BOR1;1c is critical for inflorescence development and fertility under boron limitation in Brassica napus. Plant Cell Environ..

[32] Yang L., Zhang Q., Dou J.N., Li L., Guo L.F., Shi L., Xu F.S. (2013). Characteristics of root boron nutrition confer high boron efficiency in Brassica napus cultivars. Plant Soil.

[33] Asad A., Blamey F.P.C., Edwards D.G. (2003). Effects of boron foliar applications on vegetative and reproductive growth of sunflower. Ann. Bot..

[34] Huang L.B., Ye Z.Q., Bell R.W. (1996). The importance of sampling immature leaves for the diagnosis of boron deficiency in oilseed rape (*Brassica napus* cv Eureka). Plant Soil.

[35] Skarpa P. (2013). Effect of boron foliar application at critical growth stages on sunflower (*Helianthus annuus* L.) yield and quality. J. Elem..

[36] Viçosi K.A., Carvalho A.S.d., Silva D.C., Almeida F.P., Ribeiro D., Flores R.A. (2020). Foliar fertilization with boron on the growth, physiology, and yield of snap beans. J. Soil Sci. Plant Nutr..

[37] Jankowski K.J., Sokolski M., Dubis B., Krzebietke S., Zarczynski P., Hulanicki P., Hulanicki P.S. (2016). Yield and quality of winter oilseed rape (Brassica napus L.) seeds in response to foliar application of boron. Agric. Food Sci..

[38] Huang L.B., Bell R.W., Dell B. (2008). Evidence of phloem boron transport in response to interrupted boron supply in white lupin (*Lupinus albus* L. cv. Kiev Mutant) at the reproductive stage. J. Exp. Bot..

[39] Stangoulis J.C., Brown P.H., Bellaloui N., Reid R.J., Graham R.D. (2001). The efficiency of boron utilisation in canola. Funct. Plant Biol..

[40] Liu G.-D., Wang R.-D., Wu L.-S., Peng S.-A., Wang Y.-H., Jiang C.-C. (2012). Boron distribution and mobility in navel orange grafted on citrange and trifoliate orange. Plant Soil.

[41] Hajiboland R., Farhanghi F. (2010). Remobilization of boron, photosynthesis, phenolic metabolism and anti-oxidant defense capacity in boron-deficient turnip (*Brassica rapa* L.) plants. Soil Sci. Plant Nutr..

[42] Konsaeng S., Dell B., Rerkasem B. (2010). Boron mobility in peanut (*Arachis hypogaea* L.). Plant Soil.

[43] Bergmann W. (1992). Nutritional disorders of plants: Development, Visual and Analytical Diagnosis.

[44] Hossain M.F., Pan S.G., Duan M.Y., Mo Z.W., Karbo M.B., Bano A., Tang X.R. (2015). Photosynthesis and antioxidant response to winter rapeseed (*Brassica napus* L.) as affected by boron. Pak. J. Bot..

[45] Fernández V., Eichert T. (2009). Uptake of hydrophilic solutes through plant leaves: Current state of knowledge and perspectives of foliar fertilization. Crit. Rev. Plant Sci..

[46] Miwa K., Wakuta S., Takada S., Ide K., Takano J., Naito S., Omori H., Matsunaga T., Fujiwara T. (2013). Roles of BOR2, a boron exporter, in cross linking of rhamnogalacturonan II and root elongation under boron limitation in Arabidopsis. Plant Physiol..

[47] Hegazi E.S., El-Motaium R.A., Yehia T.A., Hashim M.E. (2018). Effect of foliar boron application on boron, chlorophyll, phenol, sugars and hormones concentration of olive (*Olea europaea* L.) buds, leaves, and fruits. J. Plant Nutr..

[48] Zhao D., Oosterhuis D.M. (2002). Cotton carbon exchange, nonstructural carbohydrates, and boron distribution in tissues during development of boron deficiency. Field Crops Res..

[49] Feng Y.N., Cui R., Wang S.L., He M.L., Hua Y.P., Shi L., Ye X.S., Xu F.S. (2020). Transcription factor BnaA9.WRKY47 contributes to the adaptation of Brassica napus to low boron stress by up-regulating the boric acid channel gene BnaA3.NIP5;1. Plant Biotechnol. J..

[50] Dinh A.Q., Naeem A., Sagervanshi A., Wimmer M.A., Mühling K.H. (2021). Boron uptake and distribution by oilseed rape (*Brassica napus* L.) as affected by different nitrogen forms under low and high boron supply. Plant Physiol. Biochem..

[51] Wang S.L., Yoshinari A., Shimada T., Hara-Nishimura I., Mitani-Ueno N., Ma J.F., Naito S., Takano J. (2017). Polar Localization of the NIP5;1 Boric Acid Channel Is Maintained by Endocytosis and Facilitates Boron Transport in Arabidopsis Roots. Plant Cell.

[52] Wu X., Riaz M., Yan L., Jiang C. (2020). Distribution and mobility of foliar-applied boron (10B) in citrange rootstock under different boron conditions. J. Plant Growth Regul..

[53] Shah S., Karunarathna N.L., Jung C., Emrani N. (2018). An APETALA1 ortholog affects plant architecture and seed yield component in oilseed rape (*Brassica napus* L.). BMC Plant Biol..

[54] Livak K.J., Schmittgen T.D. (2001). Analysis of relative gene expression data using real-time quantitative PCR and the 2(T)(-Delta Delta C) method. Methods.

