# Supplementary material for: One-Time Foliar Application and Continuous Resupply via Roots Equally Improved the Growth and Physiological Response of B-Deficient Oilseed Rape
Source: Plants (Basel). 2021 Apr 26;10(5):866. doi: 10.3390/plants10050866 (PMC8146809; doi:10.3390/plants10050866)
Supplement: Supplementary file 1 [file plants-10-00866-s001.zip › plants-1179396-supplementary.pdf]

Table S1. Primers of *BnaBOR1;2* and *BnaNIP5;1* designed for real-time PCR.

Genes	Primers	Genbank accession number
BnaBOR1;2	F-5’- CCGTTGTACAGGAGATGCTTG -3’	GU827643
	R-5’- AACCCAAGAAGAAGCAAGTCG -3’	
BnaNIP5;1	F-5’- GGGGCTCACTTAAATCCATCAC -3’	KT899999
	R-5’- CCAACACTAACAGACGGAACAG -3’	
BnaActin	F-5’- TCTGGTGATGGTGTGTCTCA -3’	AF111812
	R-5’- GGTCAACATGTACCCTCTCTCG -3’	

Primer design was done by using Primer3plus and primer-blast online software.
